# Cardiac Events During Competitive, Recreational, and Daily Activities in Children and Adolescents With Long QT Syndrome

**DOI:** 10.1161/JAHA.116.005445

**Published:** 2017-09-21

**Authors:** Kristina D. Chambers, Virginie Beausejour Ladouceur, Mark E. Alexander, Robyn J. Hylind, Laura Bevilacqua, Douglas Y. Mah, Vassilios Bezzerides, John K. Triedman, Edward P. Walsh, Dominic J. Abrams

**Affiliations:** ^1^ Inherited Cardiac Arrhythmia Program & Division of Cardiac Electrophysiology Boston Children's Hospital & Harvard Medical School Boston MA

**Keywords:** arrhythmia, cardiac arrest, exercise, long QT syndrome, syncope, Ion Channels/Membrane Transport, Arrhythmias, Genetics, Electrophysiology, Sudden Cardiac Death

## Abstract

**Background:**

The 2005 Bethesda Conference Guidelines advise patients with long QT syndrome against competitive sports. We assessed cardiac event rates during competitive and recreational sports, and daily activities among treated long QT syndrome patients.

**Methods and Results:**

Long QT syndrome patients aged ≥4 years treated with anti‐adrenergic therapy were included. Demographics included mechanism of presentation, corrected QT interval pretreatment, symptom history, medication compliance, and administration of QT‐prolonging medications. Corrected QT interval ≥550 ms or prior cardiac arrest defined high risk. Sports were categorized by cardiovascular demand per the 2005 Bethesda Conference Guidelines. Each was classified as recreational or competitive. One hundred seventy‐two patients (90; 52% female) with median age 15.2 years (interquartile range 11.4, 19.4) were included. Evaluation was performed for family history (102; 59%), incidental finding (34; 20%), and symptoms (36; 21%). Median corrected QT interval was 474 ms (interquartile range 446, 496) and 14 patients (8%) were deemed high risk. Treatment included β‐blockers (171; 99%), implantable cardioverter‐defibrillator (27; 16%), left cardiac sympathetic denervation (7; 4%), and pacemaker (3; 2%). Sports participation was recreational (66; 38%) or competitive (106; 62%), with 92 (53%) exercising against the Bethesda Conference Guidelines. There were no cardiac events in competitive athletes and no deaths. There were 13 cardiac events in 9 previously symptomatic patients during either recreational exercise or activities of daily life.

**Conclusions:**

In this cohort of appropriately managed children with long QT syndrome, cardiac event rates were low and occurred during recreational but not competitive activities. This study further supports the need for increased assessment of arrhythmia risk during exercise in this patient population.

**Clinical Perspective**

**What Is New?**

- Long QT syndrome has traditionally been associated with cardiac events during exercise, leading to a restriction of competitive activities for symptomatic patients with manifest QT prolongation.
- In a cohort of appropriately treated children and young adults with long QT syndrome, recurrent cardiac event rates were low and occurred exclusively during recreational sports or activities of daily living but not during competitive activities.
- The presence of prior symptoms and longer corrected QT intervals were associated with a higher risk of recurrent cardiac events.

**What Are the Clinical Implications?**

- This study adds to the growing body of literature suggesting that competitive sports appear to carry a low risk of recurrent cardiac events among appropriately treated patients with long QT syndrome, further supporting a personalized approach in those who wish to participate in competitive sports.
- This further endorses the 2015 Bethesda Conference Guidelines permitting patients with long QT syndrome who are asymptomatic for >3 months on appropriate therapy to continue participating in competitive sports despite prior symptoms or manifest QT prolongation.

## Introduction

Long QT syndrome (LQTS) is an inherited cardiac arrhythmia characterized by delayed ventricular repolarization, manifest on the ECG as QT prolongation and/or abnormal T‐wave morphology.^1^ It has long been recognized, even from Jervell and Lange‐Nielsen's original description, that adrenergic stimulation (precipitated by exercise or emotion) further delays ventricular repolarization, which is associated with a concomitant increased risk for ventricular arrhythmias in a genotype‐specific manner.^2^ Anti‐adrenergic therapy, either β‐blockers or cardiac sympathectomy, decreases cardiac adrenergic stimulation, which in turn significantly reduces the risk of life‐threatening cardiac episodes.^3^, ^4^, ^5^

The association between adrenergic stimulation and cardiac events (CE) initially led to the development of guidelines limiting individuals to low‐intensity activities (ie, <7 metabolic equivalents [METS]) irrespective of anti‐adrenergic treatment. For example, the 2005 36th Bethesda Conference Guidelines (BCG) recommended that LQTS athletes refrain from participating in most competitive sports if they had a prior history of symptoms attributable to LQTS, QT prolongation >470 ms in males and >480 ms in females, or an implantable cardioverter‐defibrillator (ICD).^6^ The European Society of Cardiology, on the other hand, recommended avoiding competitive sports in patients with symptoms and/or corrected QT interval (QTc)>440 to 470 ms in males and 460 to 480 ms in females, those with an ICD, as well as gene carriers with no phenotypic expression.^7^ As acknowledged by the Bethesda Guidelines Expert Panel, many of the conclusions were made with a “heavy input of ‘what seems reasonable,’” noting that “decision making of this type is often faulty but is the best available.”^6^ Although exercise restriction may seem a logical approach to minimizing the risk of ventricular arrhythmias and sudden death, in reality this proves more difficult. Using accelerometers and heart rate monitors, Gow recently showed that children and adolescents frequently exceed 7 METS during free living activities.^8^ Considering the well‐recognized medical, psychological, and social benefits of competitive and recreational exercise, and the efficacy of anti‐adrenergic therapy for a significant proportion of patients with LQTS, the incidence of arrhythmic events has recently been reexamined in children and young adults with LQTS who elected to continue to engage in competitive sports against the Bethesda guidelines. Johnson reported only 1 CE in 331 athlete‐years (0.003 event rate per athlete‐year) in 60 athletes participating contrary to both Bethesda and European recommendations. This 1 event occurred in a child with marked QT prolongation (QTc>550 ms) who was noncompliant with β‐blockers.^9^ Similarly, Aziz reported no events in 12 patients participating contrary to the Bethesda guidelines.^10^ These findings led to a revision of the guidelines published in December 2015, allowing consideration of competitive sports in an athlete with either symptomatic or electrocardiographically manifest LQTS (ie, QTc >470 ms in males or >480 ms in females), after institution of treatment continued for 3 months without symptoms and with appropriate precautionary measures.^11^

In this study, we examined exertional CE rates in patients with LQTS managed at a single center; these patients participated in competitive athletics against the 2005 Bethesda recommendations. Considering the intense nature of many noncompetitive activities, we also examined the event rate during recreational activities, and compared these with the event rate during activities of daily living (ADLs).

## Methods

After obtaining Institutional Review Board approval, we invited all LQTS patients treated at Boston Children's Hospital to participate in an in‐person or telephone follow‐up interview after informed consent had been obtained. Inclusion criteria included age ≥4 years, a clinical diagnosis of LQTS made by a cardiac electrophysiologist, anti‐adrenergic therapy (β‐blockers or left cardiac sympathetic denervation [LCSD]), and regular participation in recreational or competitive exercise. Patients excluded were those with identified genetic variants in known LQTS disease‐associated genes but with no clinical evidence of QT prolongation on either resting or exercise ECG, and those with clinical or genetic evidence of Timothy or Andersen‐Tawil syndromes. Genotyping of probands and family members was performed on a clinical basis at the discretion of the treating electrophysiologist using standard disease‐specific panels performed by Clinical Laboratory Improvement Amendments (CLIA) CLIA‐approved laboratories.

All charts were retrospectively reviewed and documented for demographic, family, and clinical history. Data recorded included the following: age and mechanism of presentation (defined as incidental, family history, or cardiac symptoms); genotype; symptom history; β‐blocker type and dosage; LCSD; implantable ICD; pacemaker; and QTc.

The QT interval was manually measured in leads II or V5 as the onset of the Q wave to the end of the T wave, defined as the intersection between the T‐P isoelectric line and the downslope of the tangent of the steepest slope of the last limb of the T wave.^12^ The absolute QT interval was corrected for heart rate using Bazett's formula. The QTc was averaged over 3 to 5 consecutive heartbeats with steady R‐R interval. When available, the highest pretreatment QTc interval was used for data analysis. Those who had experienced a cardiac arrest prior to therapy and those with QTc>550 ms were considered high‐risk patients.

### CE on Anti‐Adrenergic Therapy

CE were defined as syncope, aborted cardiac arrest, appropriate discharge from ICD, or sudden death in patients managed with anti‐adrenergic therapy (β‐blockers or LCSD). Syncopal events were subdivided into “arrhythmic syncope” or “syncope of unknown etiology.” “Arrhythmic syncope” was defined as a sudden and unexpected loss of consciousness proven or believed by the managing physician to be arrhythmic in origin, while “syncope of unknown etiology” was defined as loss of consciousness without a clear arrhythmic etiology, but still concerning enough to warrant a therapy change. All syncopal episodes considered vasovagal and that did not lead to therapy modification were excluded. In the event of ICD therapies, telemetry was reviewed to assess the nature of the event and defined as appropriate (resulting from ventricular tachycardia or fibrillation) or inappropriate (resulting from supraventricular tachycardia, device or lead failure). For those with CE, the following information was retrospectively recorded and/or obtained during questionnaire interview (as described below): age, activity (competitive, recreational, or ADL); β‐blocker type and compliance for 3 days prior; administration of QT‐prolonging medications in the 24 hours preceding a CE; and subsequent therapy changes.

### Exercise and Sports

In addition to the medical history review, patients received a questionnaire about their exercise history. Prior to completing the questionnaire, patients were advised that this would not be included in their medical record and patient‐specific information would not be fed back to their primary electrophysiologist. All interviews were conducted by a single investigator (KC) who was not involved with clinical management.

Each sport/exercise was classified by cardiovascular demand, as determined by the BCG to be low (class 1A), low‐moderate (class 2A, 1B), moderate (class 3A, 2B, 1C), high‐moderate (class 3B, 2C), or high (class 3C, Figure).^13^ Sports unclassified by the BCG were assigned a classification based on the static (power) and dynamic (endurance) component. As such, yoga and marching band were classified as 1A, cheerleading and dance as 3A, and kickball as 2B. To fully capture exercise participation, we added the category of “child's play” for those whose primary exercise was general play (running, jumping, etc). Because of the wide variability in child's play, we labeled it as “recreational activity,” but did not assign it a Bethesda sport classification.

**Figure 1:**
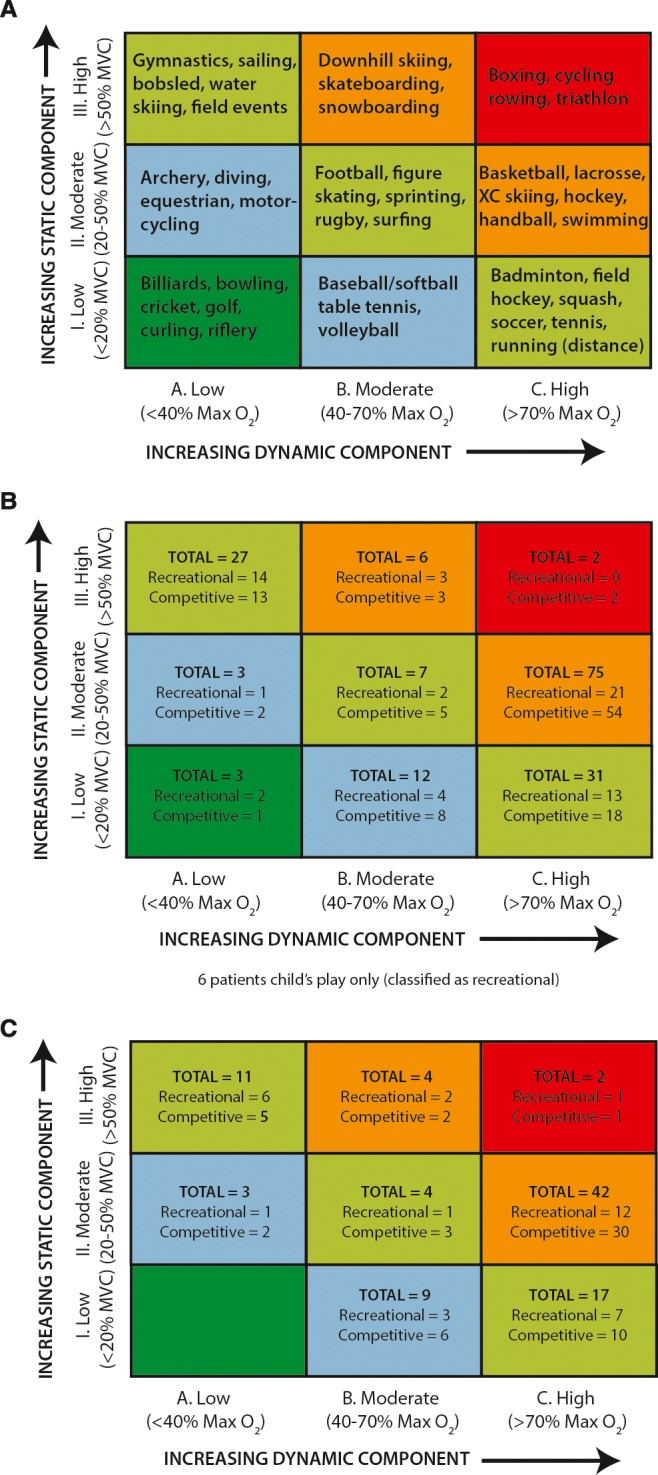
Classification of sports. A, Example of sports classified as combination of published static component (Class I–III) and dynamic component (Class A–C). Note that increasing static component is related to the estimated percent of maximal voluntary contraction (MVC) reached, resulting in an increasing blood pressure load, while increasing dynamic component is defined in terms of the estimated percent of maximal oxygen uptake (MaxO_2_) achieved, resulting in an increasing cardiac output. Adapted with permission from the 2005 AHA/ACC Eligibility and Disqualification Recommendations for Competitive Athletes with Cardiovascular Abnormalities.^13^ B, Classification of the primary sports for all patients who chose to remain active based upon the highest component sport they participate in. C, Classification of sports for those patients exercising against Bethesda recommendations.

Patients answered further questions about the sport with greatest cardiovascular demand. If a patient participated in 2 sports with similar cardiovascular demand (eg, 2 high‐moderate sports), we used the sport played most frequently for analysis. For the maximal sport, patients indicated whether participation was recreational (without regular training or competition) or competitive (with regular training and competition). They also indicated their intensity level, defined by level of perspiration and respiratory effort as low (minimal perspiration/slightly above normal breathing), moderate (definite perspiration/above normal breathing), or heavy (heavy perspiration/heavy breathing).^14^ Finally, patients indicated whether LQTS had influenced their participation in athletics.

### Bethesda Conference Guidelines (BCG)

While guidelines exist for recreational sport participation for LQTS patients,^15^ because of the often intense nature of recreational activities and to create a degree of uniformity, recreational sports were also classified as per the 2005 BCG recommendations. Moreover, the 2005 BCG guidelines were used to design this study because the 2015 American Heart Association/American College of Cardiology guidelines on LQTS sport participation were not yet published.

Therefore, patients were considered to participate against the 2005 BCG if they were active in either competitive or recreational sports higher than classification 1A and if they: were male with a QTc≥470 ms or female with a QTc≥480 ms; and/or had a prior history of cardiac arrest or arrhythmic syncope; and/or had an implantable ICD. Competitive swimmers with *KCNQ1* mutations considered disease‐causing were considered to compete against the BCG irrespective of QTc interval.

### Statistical Analysis

Study data were collected and managed using REDCap electronic data capture tools hosted at Boston Children's Hospital. Statistical analysis was performed using STATA version 13.1 (College Station, TX). Categorical variables are presented as number of patients and percentage. Continuous variables are presented as median and interquartile range (IQR) (25%, 75%). Differences in age and QTc and competitive or recreational participation according to long QT genotype were assessed using analysis of variance and *t* test, respectively. Differences in sex and symptoms by long QT genotype and participation were assessed using a χ^2^ analysis. The *t* test was used to determine the difference in QTc in those participating within and against BCG. Spearman's rank correlation was used for the comparison between perceived exertion and BCG classification. Breakthrough CE were assessed using Kruskal–Wallis test for continuous variables and Fisher exact test for categorical variables.

## Results

The case records of 276 patients in the Boston Children's Hospital LQTS database were reviewed, of whom 104 were not included on the basis of age <4 years (25), no participation in competitive or recreational activities (19), declined study participation, no response to questionnaire or inadequate information (53), patients with disease‐associated gene variants but no identifiable phenotype (4), and other conditions: Timothy syndrome (1), Andersen‐Tawil (1) and triadin knockout syndrome (1).^16^ In the 19 patients who responded but actively avoided activities, the median QTc was 460 ms (IQR 442, 469), of whom 2 were symptomatic prior to diagnosis, both with QTc intervals >500 ms (504 and 521 ms). Therefore, 172 eligible patients followed between January 1990 and May 2015 were included (Table 1).

**Table 1:** Clinical Characteristics of Study Patients

Demographics	Total=172
Sex (female/male)	90/82
Age at diagnosis, y^a^	9.0 (3.0, 13.0; range 0.08–37)
Age at study, y^a^	15.2 (11.4, 19.4; range 4.0–46.3)
QTc, ms^a^	474 (446, 496)
Treatment	
β‐Blocker only, n	142 (83%)
β‐Blocker & ICD, n	20 (12%)
β‐Blocker & PPM, n	3 (2%)
β‐Blocker & LCSD & ICD, n	6 (3%)
LCSD & ICD, n	1 (1%)
Diagnostic mechanism	
Family screening, n	102 (59%)
Incidental findings, n	34 (20%)
Syncope, n	33 (19%)
Cardiac arrest, n	3 (2%)

ICD indicates implantable cardioverter‐defibrillator; LCSD, left cardiac sympathetic denervation; PPM, permanent pacemaker; QTc, corrected QT interval.

a Values listed represent median (interquartile range; range).

The median age at time of study was 15.2 years (IQR 11.4, 19.4; range 4.0–46.3). Patients had a median QTc of 474 ms (IQR 446, 496) and were followed for a median of 7.1 years (IQR 3.5, 9.2). There was no difference in the median QTc between the study group and the 19 patients who responded and avoided exercise. The cohort included 90 females, 67 probands, and 14 high‐risk patients. All patients were receiving anti‐adrenergic therapy, in the form of β‐blocker therapy (171; 99%) or LCSD (1; 1%). In addition, 26 patients (15%) received implantable cardioverter‐defibrillators, 3 patients (2%) received permanent pacemakers, and 6 patients (3%) underwent LCSD in addition to β‐blockers. Those on β‐blocker therapy were primarily prescribed the nonselective β‐blockers, nadolol or propranolol, at 0.63 mg/kg per day (0.38, 0.87) and 1.57 mg/kg per day (0.87, 2.91), respectively (Table 2).

**Table 2:** β‐Blocker Therapy

Agent	Number	Dose, mg^a^	Dose, mg/kg Per Day^a^
Nonselective			
Nadolol	106	30 (20, 40)	0.63 (0.39, 0.87)
Propranolol	7	60 (30, 72)	1.57 (0.87, 2.91)
Selective			
Atenolol	15	37.5 (25, 50)	0.58 (0.46, 1.15)
Betaxolol	31	7.5 (5, 10)	0.16 (0.10, 0.20)
Bisoprolol	7	5 (5, 10)	0.10 (0.08, 0.15)
Metoprolol	6	25 (12.5, 25)	0.34 (0.34, 0.42)

a Values listed represent median (interquartile range).

One hundred forty patients (81%) had an identifiable mutation in 1 of the LQTS susceptibility genes, with *KCNQ1* (82; 59%), *KCNH2* (40; 29%), *SCN5A* (7; 5%), and compound mutations (8; 6%), representing the largest subsets.

### Exercise and Sports

Patients exercised on a recreational (66; 38%) or competitive (106; 62%) basis. There were no significant differences between the recreational and competitive group by QTc, prior symptom history (syncope, cardiac arrest), LQTS type, or sex (*P*>0.05); however, those in the competitive group were older (*P*<0.001) (Table 3).

**Table 3:** Baseline Demographic Variables and Prior Symptoms by Genotype and Athletic Participation

	*KCNQ1*	*KCNH2*	*SCN5A*	Multiple	*P* Value	Competitive	Recreational	*P* Value
Number	82	40	7	8		106	66	
Age, y	15.8 (11.6, 20.4)	14.2 (9.7, 17.5)	12.4 (10.7, 15.8)	14.3 (13.5, 16.9)	0.26	16.3 (12.8, 19.8)	12.8 (8.0, 17.3)	0.0003
QTc, ms	455 (443, 488)	476 (448, 516)	461 (443, 498)	496 (460, 549)	0.006	466 (443, 501)	472 (447, 489)	0.84
Sex (female)	39	21	3	5	0.82	60	30	0.16
Asymptomatic	64	32	4	3	0.04	32	17	0.32
Prior symptom:								
Syncope	18	7	3	5	0.03	27	11	0.18
Cardiac arrest^a^	0	1	0	0	0.49	1	2	0.31
*KCNQ1*						50	32	0.28
*KCNH2*						27	13	0.84
*SCN5A*						6	1	0.42
Multiple						6	2	0.71

LQTS indicates long QT syndrome; QTc, corrected QT interval.

a Please note that a total of 3 cardiac arrests occurred in the cohort prior to LQTS diagnosis, 2 of which occurred in genotype‐negative patients. Values are listed as number or median (interquartile range) as appropriate.

Patients participated mostly in moderate (class 1C, 2B, or 3A) or high‐moderate (3B or 2C) sports (Figure). Where possible, each participant recorded his or her perceived intensity level as light (14; 19%), moderate (24; 34%), or heavy (34; 47%). When comparing patient‐perceived exertion with BCG‐classified exertion level, we observed overall good agreement between the 2 methods (Spearman *r*=0.69). There was no significant difference in median (IQR) QTc interval related to patient‐perceived intensity level; light 481 ms (477–504); moderate 477 (445–507); heavy 482 ms (455–497) (*P*=0.23), although those with prior symptoms were more likely to undertake a lighter intensity level of activity (light 44%; moderate 15%; heavy 13%) (*P*=0.01).

Out of 72 respondents asked if a diagnosis of LQTS had influenced them to stop or not take up a specific sport, 37 (51%) replied no and 35 (49%) replied yes. There was no significant difference between the 2 groups in terms of QTc interval (477 ms [454–491] versus 478 ms [451–499], *P*=0.85) or prior symptoms (16% versus 26%, *P*=0.32).

### Bethesda Conference Guidelines (BCG)

Ninety‐two patients (53%; 47 female) participated in sports against the BCG on a recreational (33) or competitive (59) basis, primarily in high‐moderate sports (Figure), including 50 probands, and 12 high‐risk individuals, with a median age of 15.5 years (11.9, 20.7) and follow‐up of 7.2 years (3.4, 11.3). Only 1 patient with a *KCNQ1* mutation (long QT1) and normal QT interval (QTc 451 ms) was involved in competitive swimming. Those who participated in athletics against BCG had a longer QTc than those who participated in accordance with guidelines (491 ms [474, 513] versus 447 [436, 459], *P*<0.05).

### Cardiac Events

In a combined 1203 years of patient follow‐up, there were 13 CE in 9 patients (Tables 4 and 5), 8 of whom were symptomatic prior to their diagnosis of LQTS, and had significantly longer QT intervals than those patients who remained asymptomatic (Table 6). Four events occurred during recreational exercise and 9 during ADL. There were no deaths.

**Table 4:** CE During Recreational Activities

Patient	Sex	QTc, ms^a^	Initial Symptom	Genotype	Age, y	Exercise (Bethesda class)^b^	QT^+^Rx^c^	β‐Blocker	ICD	Outcome
#1	Male	507	Exertional syncope	*KCNQ1*	15	Skateboarding (IIIB)	No	Nadolol (compliant)	No	ICD implanted
#2	Male	498	Exertional cardiac arrest	Negative	12	Long distance running (1C)	No	Nadolol (compliance unknown)	Yes—ICD shock	Increased BB dose
#3^d^	Female	500	Exertional syncope	Negative	10	Child's play	No	Atenolol (compliant)	No	ICD implanted; BB changed
#4	Male	528	Exertional syncope	*KCNQ1*	10	Child's play	No	Nadolol (compliance unknown)	No	ICD implanted

BB indicates β‐blocker; CE, cardiac events; ICD, implantable cardioverter‐defibrillator.

a Highest pretreatment corrected QT interval reported.

b Reported exercise at the time of cardiac event.

c QT^+^Rx, QT‐prolonging medications.

d Patient #3 is the same as patient #1 in Table 5.

**Table 5:** CE During ADL

Patient	Sex	QTc, ms^a^	Initial Symptom	Genotype	Age, y	Activity^b^	QT^+^Rx^c^	β‐Blocker	ICD	Outcome
#1^d^	Female	500	Exertional syncope	Negative	10	Walking in a winter storm	No	Nadolol (compliant)	Yes—3 shocks for TdP	Mexiletine added
#2	Female	513	Syncope	*KCNH2*	17	Walking	No	Nadolol/labetalol (compliant)	No	ICD implanted
					18	Auditory stimulation	No	Nadolol/labetalol (compliant)	Yes—1 shock for TdP	BB dose increased
#3	Female	450	Syncope	*KCNQ1*	23	Using copy machine	No	BB unknown (noncompliant)	No	ICD implanted
#4	Female	590	Syncope	*KCNH2*	14	Giving speech at school	No	Nadolol (compliance unknown)	Yes—TdP self‐terminated	Mexiletine added
#5	Female	585	Syncope	*KCNQ1*	4	Shopping	Unknown	Atenolol (noncompliant)	Yes—TdP storm^e^ with ICD shock	Change BB
					5	Using bathroom	Unknown	Nadolol (compliance unknown)	Yes—TdP storm with ICD shock	LCSD
#6	Male	553	TdP during surgery	*KCNH2*	7	Running from car to garage	No	Propranolol (compliant)	Yes—TdP storm with ICD shock	Changed BB
					8	Post ICD revision	No	Atenolol (compliant)	Yes—TdP storm with ICD shock	Changed BB

ADL indicates activities of daily living; BB, β‐blocker; CE, cardiac events; ICD, implantable cardioverter‐defibrillator; LCSD, left cardiac sympathetic denervation; QTc, corrected QT interval; TdP, torsades des pointes.

a Highest pretreatment QTc reported.

b Reported activity at the time of cardiac event.

c QT^+^Rx, QT prolonging medications.

d Patient #1 is the same as patient #3 in Table 4.

e TdP storm defined as 3 or more ICD therapies within a 24‐h period.

**Table 6:** CE During Daily Living and Recreational Activities

	No CE	CE During ADL	CE During Recreational Sports	*P* Value
Number	163	6	4	
Age at diagnosis, y^a^	9 (3, 13)	8 (5, 10)	8 (7, 10)	0.97
Prior symptoms	20%	83%	100%	<0.01
QTc, ms^a^	466 (445, 494)	533 (468, 577)	503 (487, 512)	0.03
Genotype				0.52
*KCNQ1*	50%	17%	50%	
*KCNH2*	22%	33%	···	
*SCN5A*	4%	···	···	
Multiple	4%	17%	···	
Negative/unknown	20%	33%	50%	

ADL indicates activities of daily living; CE, cardiac events; QTc, corrected QT interval.

a Values listed represent median (interquartile range).

### CE During Exercise

There were no syncopal events reported during competitive exercise, and no cardiac arrests or deaths during either recreational or competitive exercise. Four patients experienced a single episode of syncope during recreational exercise, which in 1 case led to an appropriate ICD therapy. All 4 patients participated in sports categorized >1A, and 1 patient was considered high risk (by virtue of prior cardiac arrest). One event occurred during skateboarding (high‐moderate), 1 during long‐distance running (moderate), and 2 during child's play (Table 4). In 2 patients, β‐blocker compliance was confirmed and in the other 2, this was unknown. None of the 4 patients had received QT‐prolonging medications.

### CE During ADL

Six patients (3 high risk by virtue of QTc>550 ms) experienced 9 CE during ADL (Table 5). Patients were compliant with β‐blockers on 5 occasions, noncompliant on 2 occasions, and unknown on 2. No event occurred within 24 hours of taking known QT‐prolonging medications, although 1 patient was receiving an unknown antibiotic.

## Discussion

This single‐center study provides further documentation of the rate of CE in an appropriately managed cohort of patients with LQTS. None of the 59 patients who continued to participate competitively had a CE. Four episodes of arrhythmic syncope occurred during recreational activity (including 2 during child's play) and a further 9 episodes during ADL. All those who had symptoms on anti‐adrenergic therapy were symptomatic prior to treatment, which has important implications for the ever‐increasing number of asymptomatic patients diagnosed by clinical or genetic cascade familial screening. These data support that of Johnson^9^ and Aziz^10^ in describing event rates significantly lower than initially anticipated, and enhances the notion that many appropriately counseled and managed children and young adults with LQTS choose to continue competitive activities, and do so safely. These findings have been reflected in the most recent Bethesda guidelines published in 2015, permitting symptomatic patients with LQTS who are asymptomatic for >3 months on appropriate therapy to continue participating competitively despite prior symptoms or manifest QT prolongation. Fundamental to such an approach, however, is the understanding from the whole family that a small but undefinable risk persists, that medication compliance and avoidance of QT‐prolonging agents is mandatory, and that coaches and school staff accept the athlete's participation and that the appropriate resuscitation equipment and those trained in its use are present during sporting activities.

### CE During Exercise

While fully respecting the importance of the BCG, our institutional approach was to allow appropriately managed and counseled patients to continue activities following a diagnosis of long QT syndrome, with 59 patients participating in regular competitive exercise against the recommendation of the Bethesda guidelines, and 33 continuing in recreational activities. Of those who regularly exercised, only 3 participated in approved class 1A sports; instead, most were involved in activities requiring moderate or high‐moderate cardiovascular demand. Even though most patients participated in competitive sports, which are commonly presumed to be more demanding and intense, all 4 of the exercise‐precipitated syncopal events occurred during recreational activities. Although this may suggest recreational and competitive activities may be equally intense, it is of course possible that these individuals were of higher risk by virtue of prior symptomatic presentation and therefore limited themselves by choice. Similarly, a number of patients at potentially higher risk may have decided to not participate in any activities, thereby invoking an element of selection bias, and 67 patients in our institutional cohort were excluded from the study on that basis.

The Bethesda guidelines restricted competitive athletes under the reasonable assumption that competitive sports are more intense and therefore confer greater cardiac risk; however, the relationship between intensity and cardiac risk in appropriately managed LQTS patients remains unclear. Defining causality between levels of intensity and arrhythmic risk is challenging given the significant personal variability in different genetic, phenotypic, and environmental factors, and makes it challenging to use “intensity levels” to design exercise restrictions. Depending on motivation, skill level, and sport type, what is recreational for 1 patient may be intensely competitive for another.

### CE During ADL

During daily activities, children and adolescents frequently exceed the 7 METS limit set by BCG/ESC.^8^ In this study, there were 9 CE during ADL: 8 occurred during activities requiring <7 METS (walking to class, using the copy machine, etc) and 1 occurred during an activity requiring >7 METS (walking vigorously through a snowstorm). Overall, therefore, the 8 CE during daily activities are only marginally greater than the 5 events reported during exercise. It is estimated that children and adolescents spend ≈90% of time performing activities that require <7 METS energy expenditure.^8^ Therefore, the proportional event rate may be significantly higher in this experience during exercise and more exertional daily activities. Children typically spend 4% of their day (7 hours/week) engaged in exertional activities,^17^ and a further 2.3% above the 7 MET threshold daily activities. Based on our follow‐up of 1203 patient‐years, this translates approximately to 49 patient‐years follow‐up during exercise, 28 patient‐years follow‐up during ADL>7 METS, and 1143 patient‐years follow‐up during ADL<7 METS. As such the proportional CE rates are highest during exercise 0.08 events/year (although none during competitive exercise), compared with 0.04 and 0.007 events/year during ADL of >7 and <7 METS, respectively. This again may reflect the specific risk profile of the individual patients, although limiting children and adolescents from child's play or walking in a storm would prove very challenging.

### Comparative CE Rates

CE rates in this experience were similar to those recently reported during competitive exercise. In a study published in 2013, Johnson reported an event rate of 0.003 event/year among 60 athletes participating contrary to both the Bethesda and European recommendations,^9^ while Aziz in 2015 reported no events in 12 patients participating contrary to the Bethesda guidelines.^10^ Overall, these CE rates are significantly lower than that previously published,^18^, ^19^ which may be explained in a number of different ways.

Firstly, a large proportion of this cohort (67%) was asymptomatic when diagnosed with LQTS, identified through family screening or because of an incidental electrocardiographic finding of QT prolongation. Increasingly, evidence suggests those who are asymptomatic at diagnosis remain so once started on appropriate β‐blockers^3^, ^20^. The increasing recognition of LQTS in the medical community and the implementation of familial screening means that many are diagnosed prior to the onset of symptoms, compared with prior eras when more than half of patients were diagnosed following a symptomatic event.^3^, ^18^, ^19^, ^21^ Secondly, and closely associated with the above, the overall QTc intervals reported here are shorter than prior studies, whose average QTc range up to 520 ms. Therefore, there is a higher proportion of asymptomatic patients in this category with overall less QT prolongation, suggesting an overall lower risk profile when compared with historical cohorts. Finally, we attempted to differentiate between different syncopal events, excluding those not considered to be arrhythmic in nature, which may further reduce the measured event rate. Given the very high incidence of benign syncope in teenage life affecting 50% of females and 20% of males,^22^ we included only events considered to be arrhythmic at the time by the managing physician.

### Limitations

With this observational single‐center retrospective study, we were limited to data from medical records and from follow‐up interviews, which may introduce recall bias. Importantly, as already mentioned, a significant proportion of patients managed in this institution were not involved in either recreational or sporting activities, which may represent self‐exclusion based on a higher risk profile, and therefore not be truly representative of the entire experience of CE occurring during activities of daily life. Moreover, we did not describe CE for patients exercising off anti‐adrenergic therapy because almost all patients at Boston Children's Hospital are managed with anti‐adrenergic therapy. In addition, there are a number of sports in the study that were poorly represented. Accordingly, we did not risk assess different sports and sport levels. Results should not be extrapolated to all sports and competition levels. Finally, with an overall low CE rate in this cohort, the current analysis may have lacked adequate power to examine differences in CE rates based on compliance with exercise restriction. The results nevertheless add to others in the literature, which have observed similar findings^9^, ^10^ and taken together, support a growing body of literature in this field.

## Conclusions

In 1203 years of patient follow‐up, we report a low CE rate among appropriately managed patients with LQTS. Importantly, there were no events during competitive athletics in the 59 patients who elected to continue against the 2005 Bethesda guidelines. All events occurred during recreational athletics or activities of daily life.

While the CE rate during exercise is proportionally greater than during ADL, the event rate during exercise is still very low. Our CE rate during exercise reflects data published from other institutions^9^, ^10^ and further supports the importance of patient and family choice coupled with expert counseling in decisions regarding the continuation of sporting activities. While it is necessary to account for risks during exercise, it is still important to recognize the positive social, emotional, and health benefits of a regular exercise program. We believe LQTS patients seeking an active lifestyle should be provided with appropriate data and counseling to balance the relative risks and benefits of continued participation. The ongoing National Institutes of Health funded prospective study to determine how lifestyle and exercise impact the well‐being of individuals with LQTS (www.livelqts.org) will undoubtedly provide further information on the safety and benefit of sporting activities in patients with long QT syndrome.

## Sources of Funding

The Inherited Cardiac Arrhythmia Program is generously supported by the Mannion and Roberts families.

## Disclosures

None.
